# Integrating deep learning architectures for enhanced biomedical relation extraction: a pipeline approach

**DOI:** 10.1093/database/baae079

**Published:** 2024-08-28

**Authors:** M Janina Sarol, Gibong Hong, Evan Guerra, Halil Kilicoglu

**Affiliations:** Informatics Programs, University of Illinois Urbana-Champaign, 614 E Daniel Street, Champaign, IL 61820, United States; School of Information Sciences, University of Illinois Urbana-Champaign, 501 E Daniel Street, Champaign, IL 61820, United States; School of Information Sciences, University of Illinois Urbana-Champaign, 501 E Daniel Street, Champaign, IL 61820, United States; School of Information Sciences, University of Illinois Urbana-Champaign, 501 E Daniel Street, Champaign, IL 61820, United States

## Abstract

Biomedical relation extraction from scientific publications is a key task in biomedical natural language processing (NLP) and can facilitate the creation of large knowledge bases, enable more efficient knowledge discovery, and accelerate evidence synthesis. In this paper, building upon our previous effort in the BioCreative VIII BioRED Track, we propose an enhanced end-to-end pipeline approach for biomedical relation extraction (RE) and novelty detection (ND) that effectively leverages existing datasets and integrates state-of-the-art deep learning methods. Our pipeline consists of four tasks performed sequentially: named entity recognition (NER), entity linking (EL), RE, and ND. We trained models using the BioRED benchmark corpus that was the basis of the shared task. We explored several methods for each task and combinations thereof: for NER, we compared a BERT-based sequence labeling model that uses the BIO scheme with a span classification model. For EL, we trained a convolutional neural network model for diseases and chemicals and used an existing tool, PubTator 3.0, for mapping other entity types. For RE and ND, we adapted the BERT-based, sentence-bound PURE model to bidirectional and document-level extraction. We also performed extensive hyperparameter tuning to improve model performance. We obtained our best performance using BERT-based models for NER, RE, and ND, and the hybrid approach for EL. Our enhanced and optimized pipeline showed substantial improvement compared to our shared task submission, NER: 93.53 (+3.09), EL: 83.87 (+9.73), RE: 46.18 (+15.67), and ND: 38.86 (+14.9). While the performances of the NER and EL models are reasonably high, RE and ND tasks remain challenging at the document level. Further enhancements to the dataset could enable more accurate and useful models for practical use. We provide our models and code at https://github.com/janinaj/e2eBioMedRE/.

**Database URL**: https://github.com/janinaj/e2eBioMedRE/

## Introduction

Biomedical publications are a rich and valuable source of scientific knowledge. Keeping up with the ever-growing literature and generating insights from it is an essential yet challenging task in the workflows of researchers and other stakeholders of biomedical science. Automated methods for extracting and organizing the literature knowledge could improve the efficiency of such workflows and expedite the construction of knowledge bases, accelerating scientific discovery and enhancing understanding of disease and health [1]. Natural language processing (NLP) and text mining methods have long been proposed for information extraction from the literature, and substantial progress has been made over the last two decades in tasks such as named entity recognition (NER) and relation extraction (RE) [2]. Despite the progress in certain areas, the use of biomedical NLP tools in real-world applications remains limited, partly due to modest performance and methods that do not generalize well [3].

Shared task competitions, such as BioCreative challenges [4, 5] and BioNLP event extraction shared tasks [6, 7], have stimulated much of the progress made in biomedical NLP by providing sizable benchmark corpora and bringing together communities to improve the state-of-the-art in specific NLP tasks. BioCreative, launched in 2004, has been one of the long-term, sustained efforts in advancing biomedical NLP through challenges. It has led to the development of many datasets and NLP approaches over the years that aim to address various information extraction tasks related to biology literature, including gene mention identification [8] and extraction of chemical-induced diseases [9]. In its latest edition (BioCreative VIII), one of the shared tasks (BioRED Track) focused on biomedical NER and RE, as well as named entity linking (EL) and novelty detection (ND). This track expands upon the earlier BioCreative competitions that addressed one or a small number of entity and relation types [8, 9] by considering the BioRED dataset [10], which includes six entity types (Disease, Chemical, Gene, Species, Cell Line, and Variant) and eight relation types that hold between these entity types (Association, Positive Correlation, Negative Correlation, Bind, Comparison, Conversion, Cotreatment, and Drug Interaction). The novelty of each relation is also considered. Extracting such knowledge from the literature could support many downstream tasks, such as biocuration [11], pharmcovigilance [12], and literature-based discovery [13].

In this study, we build on our submission to BioCreative VIII BioRED Track, which has demonstrated competitive performance [14], and present an enhanced end-to-end system that performs all four tasks in a pipeline architecture. For each task, we enhance our previous approach or experiment with alternative approaches. Specifically, for NER, we compare our token classification model based on Bidirectional Encoder Representations from Transformers (BERT) to a BERT-based span classification model, and retrain the better-performing model by incorporating additional datasets into the training process. For EL, we use a hybrid approach that includes a convolutional neural network (CNN) for disease and chemicals [15], and uses an external entity linker, PubTator 3.0 [16], for other types. For RE and ND, we extend the BERT-based Princeton University Relation Extraction system (PURE) model [17] to deal with document-level relations. The combination of token classification model for NER, CNN-augmented EL, and modified PURE model for RE and ND yields our best overall results, showing a substantial improvement over the shared task submission. Using additional datasets in the training process seems to account for most of the performance improvements.

## Related work

NER and RE from biomedical literature are foundational tasks in biomedical NLP. Some established systems aim to provide broad coverage of NER and RE. They have focused on rule-based methods and leveraged rich semantic resources, such as Unified Medical Language System [18], to extract concepts and semantic relation triples from the biomedical abstracts [19, 20]. Most current NER and RE approaches are based on supervised machine learning and are trained on corpora that often include a small number of entity or relation types, which limits their usefulness for practical purposes [10]. In recent years, deep learning architectures have led to state-of-the-art performance on biomedical NER and RE corpora. These methods often involve fine-tuning domain-specific pretrained language models based on Transformer architecture, such as BioBERT [21] and PubMedBERT (also known as BioMedBERT) [22], on corpora annotated for biomedical entities [23, 24] and relations [25, 26]. As these corpora are limited in scope, broad-coverage, rule-based tools, such as MetaMap [19] and SemRep [20], despite their modest performance, have remained popular for downstream tasks in the biomedical domain. More recently, in-context learning based on generative large language models (LLMs), such as GPT-3, has also been explored, although these methods generally underperform fine-tuning approaches [27–29].

Current methods for biomedical NER typically use a token classification approach based on the BIO scheme or formulate NER as a span classification task, where spans up to a particular length are considered independently as input [30]. State-of-the-art results on NER benchmark datasets have been reported using domain-specific BERT models [22, 31]. It has been acknowledged that models trained on a single dataset do not generalize well [3, 32], and various methods, such as multi-task learning on several datasets [33], have been explored to address this problem. In contrast, Luo *et al*. [32], in recent work, merged multiple datasets into a single sequence labeling task via task-oriented tagging labels and using a PubMedBERT-CRF model to obtain state-of-the-art NER results on the BioRED dataset [10].

Supervised biomedical RE is generally formulated as a binary or multi-class classification task, where domain-specific BERT models provide the foundation for classification of entity pairs into one of the predefined relation types [2]. Training corpora for biomedical RE often focus on one or a small number of relation types [25, 26, 34]. BioRED [10] is more comprehensive and includes six entity and eight relation types, although some of these relation types are rare in the dataset. Lai *et al*. [35] combined and aligned several RE datasets with BioRED and have shown improvement in model performance, particularly for the rare labels. While RE is often performed in a pipeline approach where NER is followed by RE [17], joint learning approaches have also been explored [15, 36, 37].

EL is a particularly important task in biomedical NLP, as it normalizes entity mentions to standard concept identifiers in knowledge bases and helps aggregate the evidence across documents [38]. Early work on biomedical EL focused on rule-based approaches [19, 39, 40], which are efficient and interpretable, but generally underperform more recent supervised machine learning approaches, such as DNorm [41]. With the advent of deep learning, neural network architectures, such as CNN-based ranking [42], Bidirectional Long Short-Term Memory [43], and BERT-based models [44, 45], have been applied to biomedical EL, yielding improved performance.

Some web-based tools and Application Programming Interfaces have enabled rapid NER and EL for biocuration of scientific articles. For example, PubTator [11] provides easy access to named entities and their database identifiers in PubMed abstracts, and has been extended to full-text articles in PubMed Central (PubTator Central [46]) and relations (PubTator 3.0 [16]). These tools focus on a limited set of entity and relation types.

## Methods

**Figure 1.**
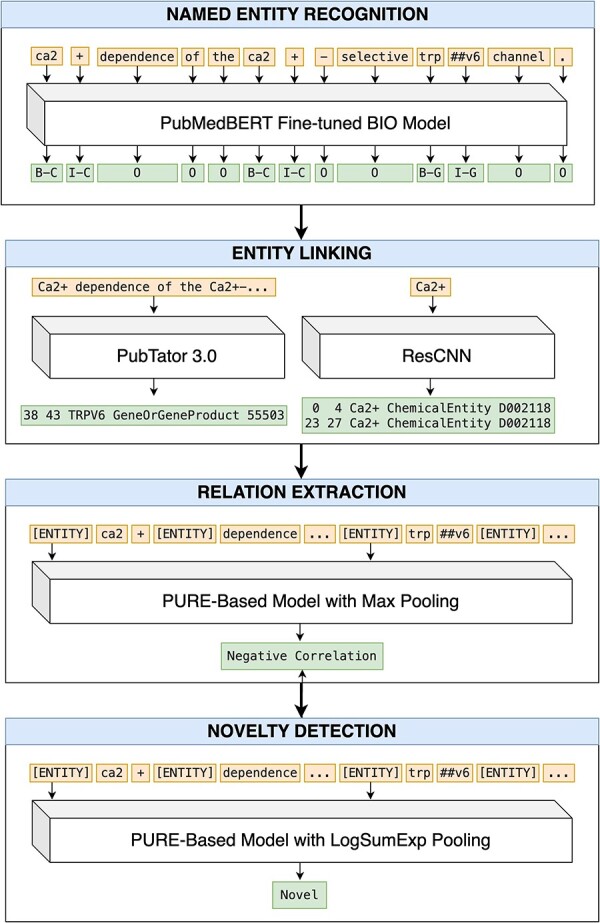
An end-to-end pipeline for RE and ND. Article title: Ca2+ dependence of the Ca2+-selective TRPV6 channel (abstract omitted for clarity).

We propose a pipeline approach for our end-to-end RE system, where each of the four tasks (NER, EL, RE, and ND) are performed sequentially (Fig. 1). We describe our approach for each step below.

### Dataset

We primarily use the BioRED dataset [10, 47] for training and evaluation. The latest version of the BioRED dataset [47], in the BioCreative VIII BioRED Track, contains a total of 1000 PubMed abstracts split into training (600) and test sets (400). We split the training set in accordance with the track; 500 abstracts are used as the training set and 100 abstracts serve as the development set.

Table 1 shows the distribution of entity mentions for each data split. The training set contains 3280 unique entities (based on controlled vocabulary identifiers): 1379 (42.04%) are genes, 675 (20.58%) are diseases, 580 (17.68%) are chemicals, 546 (16.65%) are sequence variants, 43 (1.31%) are species, and 57 (1.74%) are cell lines. The development set contains 985 unique entities of which 400 (40.61%) are genes, 245 (24.87%) are diseases, 171 (17.36%) are chemicals, 137 (13.91%) are sequence variants, 11 (1.12%) are species, and 21 (2.13%) are cell lines. Test set annotations are not publicly available; therefore, we use the counts from the shared task overview paper [47], which does not provide unique entity counts.

**Table 1.** Distribution of entity mentions in the BioRED dataset. Knowledge base refers to the vocabulary used for grounding the entities of specific types

Entity type	Knowledge base	Train	Dev	Test	Total
Gene	NCBI Gene [62]	5517	1180	5728	12 425
Disease	MEDIC [56]	4628	917	3641	9186
Chemical	MESH [63]	3675	754	2592	7021
Species	NCBI Taxonomy [64]	1799	393	1774	3966
Variant	dbSNP [65]	1140	241	1525	2906
Cell line	Cellosaurus [66]	125	50	140	315
**Total**		**16 884**	**3535**	**15 400**	**35 819**

Table 2 shows the distribution of relation types for the training, development, and test sets. Associations, positive correlations, and negative correlations make up 95.74% of all relations. Other relation types are rare in the dataset. It is important to also note that BioRED relations do not have directionality, in contrast to most other RE models.

**Table 2.** Distribution of relations by type in the BioRED dataset

Relation type	Train	Dev	Test	Total
Association	2752	635	2759	6146
Positive correlation	1441	325	1751	3517
Negative correlation	979	171	1192	2342
Cotreatment	41	14	172	227
Bind	80	9	136	225
Comparison	33	6	13	52
Conversion	3	1	13	17
Drug interaction	11	2	0	13
**Total**	**5340**	**1163**	**6036**	**12 539**

### Named entity recognition

#### BERT-based token classification model

We first framed the NER task as a token classification problem, utilizing the BIO scheme to mark each token as starting (B-), inside (I-), or outside of an entity (O), with corresponding type information such as B-Chemical and I-CellLine. We fine-tuned the pre-trained BioMedBERT model [22] and used softmax for predicting token labels. The maximum sequence length is 512 tokens. Cross-entropy was used as the loss function. We performed grid search to optimize hyperparameters: learning rate (1e-04, 2e-04, 3e-04, 1e-05, 2e-05, 3e-05, 1e-06, 2e-06, 3e-06), batch size (8, 16), and epochs (1–50). The optimal hyperparameter combination was found to be learning rate (3e-05), batch size (16), and epochs (22). The training and development sets described above were used for training and evaluation, respectively.

Inspired by the finding that combining multiple biomedical NER datasets can improve model performance [32], we also sought to incorporate multiple annotated datasets during training. We used the same datasets as All-in-one Named Entity Recognition (AIONER) [32], as our focus is on the same entity types: GNormPlus [48] and NLM-Gene [49] for genes; NCBI Disease [23] and BC5CDR [25] for diseases; NLM Chem [49] and BC5CDR [25] for chemicals; Species-800 [50] for species; BioID [51] for cell lines; and tmVar3 [52] for variants. We excluded Linnaeus [53], a species dataset, because it includes full text publications, which exceed the maximum sequence length, and lower F_1_ score was reported with the addition of this dataset [32]. This resulted in a total of 9 datasets, including the BioRED dataset. Both training and test sets of these additional datasets were used for training the final model.

With the exception of BioRED, which has annotations for all entity types, and BC5CDR, which has chemical and disease annotations, all other datasets include a single entity type. This may confuse the model, as relevant entity mentions may be unannotated in a dataset that does not focus on that entity type. Consider the following example from the GNormPlus dataset: ‘Germline mutations of the human BRCA2 gene confer susceptibility to breast cancer.’ As GNormPlus includes gene annotations only, the disease ‘breast cancer’ is not annotated. To mitigate this issue, we employed the approach outlined in Luo *et al*. [32], by enclosing the input sentence in special tokens to indicate the entity types for which the model should generate predictions (e.g. <ALL>-</ALL>, <GENE>-</GENE>, <CHEMICAL-DISEASE> </CHEMICAL-DISEASE>). In addition, instead of using a single outside token (O), we also used specific type information (e.g. O-ALL, O-GENE, O-CHEMICAL-DISEASE). The ALL designation is reserved for the BioRED dataset, which contains annotations for all entity types.

#### PURE span classification model

We also explored a span classification approach to NER. This involves passing token spans, up to a predefined length, to the model for named entity type classification. For this task, we leveraged an existing NER model, PURE [17], which uses BERT to generate contextualized token representations. The contextualized embeddings of candidate token spans are then fed into a two-layer feedforward network for classification. We set the maximum token span length to 30, reflecting the maximum entity span length in the training set. Additionally, we conducted hyperparameter tuning, exploring various learning rates (1e-04, 2e-04, 3e-04, 1e-05, 2e-05, 3e-05, 1e-06, 2e-06, 3e-06) and number of epochs (1–50). We identified that a learning rate of 1e-05 and 39 epochs yields the best F_1_ score on the development set. Throughout our experiments, we kept the batch size constant at 32.

#### Post-processing named entity predictions

Lastly, to further improve predicted entities, we applied the following post-processing rules:

1. If two entity mentions of the same type appear consecutively with no whitespace in between, we combine them into a single entity mention (e.g. ‘A’ and ‘(1)-adenosine receptor’ vs ‘A(1)-adenosine receptor’).
2. If two disease entities are separated by a single character that is not a slash (/), we combine them (e.g. ‘benign’ and ‘tumor’ vs ‘benign tumor’).

### Entity linking

We initially experimented with different existing approaches to EL: PubTator Central [46], BERN2 [54], and PubTator 3.0 [16]. Overall, PubTator 3.0 performed better than the other methods. However, we also recognized that there was room for improving upon PubTator 3.0, especially with Disease and Chemical entities. Improvement for these entity types could also significantly impact the downstream RE task, as most relations in the dataset involve Disease and Chemical entities. For these two entity types, we trained relatively lightweight CNN models with residual connections (ResCNN) [15], as described below.

#### ResCNN for disease and chemical entity linking

A typical EL approach is to train an encoder module which encodes entity mentions and the entity names from controlled vocabularies to the same embedding space, and then to use encoded query entity mentions to retrieve similar entity names from the vocabulary based on a similarity metric, such as cosine similarity.

To improve the EL performance for Disease and Chemical entities, we employ ResCNN [15] as our encoder. This architecture was motivated by the observation that, even when the order of input tokens is shuffled or attention scope is limited, the performance of a BERT-based EL model is nearly identical, which suggests that a CNN model that captures the local interactions might perform just as well as BERT-based models. ResCNN has been shown to perform comparably to BERT-based EL models, while using 1*/*100 of the parameters in BERT-based models [44, 45].

ResCNN-based EL architecture consists of a token embedding layer, an encoding layer, and a pooling layer. The token embedding layer tokenizes the input and initializes the embeddings using a BERT-based model’s contextualized embeddings, which are frozen during training. The encoding layer includes multiple blocks with convolutional filters of varying sizes [55]. A position-wise fully connected feed forward network and a residual connection are applied to each block. Lastly, max pooling [55] is used for the pooling layer to obtain the final vector representations. We conducted a greedy search to tune some hyperparameters for each model (Table 3). For other hyperparameters, we used the default ResCNN settings: 300 filters for the convolutional network, and 100 epochs for training with the evaluation for every 5 epochs.

**Table 3.** Search space for hyperparameter tuning of ResCNN-based models for disease and chemical mentions

	Search space
Pooling type	Max^+^, Mean, Attention*
Learning rate	1e-3*^+^, 5e-3, 1e-4, 3e-4, 5e-4
# Encoder	3^+^, 4*, 5
Feature size	128, 256*^+^, 512
Dropout rate	0.1, 0.25*^+^, 0.5

* Denotes the optimal hyperparameter for ResCNN-Disease model, and

^+^denotes the optimal hyperparameter for ResCNN-Chemical model.

We used the latest versions of Merged Disease Vocabulary [56] and Comparative Toxicogenomics Database (CTD) [57] to extract synonym-ID pairs and build indexes for Disease and Chemical concepts, respectively. To fully leverage the ID-mention pairs from the training set, we also added them to the index before evaluating EL on the development set. In addition, to train the models for Disease and Chemical concepts, we augmented BioRED with NCBI Disease corpus [23] and BC5CDR corpus [25], respectively. We also searched the optimal choice for ResCNN initial vector representation by initializing with BioMedBERT (same as PubMedBERT) [22] and BioLinkBERT [31] embeddings. We report the EL performance using different training sets (original vs augmented) and embedding layers (BioMedBERT vs BioLinkBERT).

In line with previous work [15, 39, 44], we use top-k accuracy to report task-specific performance for ResCNN-based EL models. We note that the EL evaluation in BioCreative VIII BioRED Track is conducted at the document level by matching (PMID, entity type, id) tuples. If a mention is mapped to multiple identifiers, they are considered multiple tuples.

#### PubTator 3.0

For other remaining entity types (Gene, Species, Variant, Cell Line), we simply leveraged PubTator 3.0 [16]. PubTator 3.0 uses AIONER [32] for NER and normalizes predicted mentions using GNorm2 [58] for genes and species, tmVar3 [52] for variants, and TaggerOne [59] for cell lines. As we use our own NER modules, some PubTator entity mention spans do not exactly align with our predicted mention spans. To overcome this issue, we allow partial matching in order to fully leverage the normalization predictions from PubTator 3.0. Furthermore, we also build a look-up dictionary with the PubTator 3.0 predictions so that we can also normalize entity mentions which the ResCNN-based method is unable to resolve to a concept identifier.

### Relation extraction

We adapted the PURE model [17], originally comprising two separately fine-tuned BERT models for NER and RE. We utilized the RE model, which uses the generated entity representations for labeling entity pairs with a relation type (or no relation). For this purpose, all tokens belonging to an entity mention are enclosed with marker tokens denoting the entity type and whether the entity is the subject or object of a relation [e.g. (SUBJ:GENE)]. For each entity, the embedding of its corresponding marker token (from the last hidden state of the BERT model) is taken as its representation. The embeddings of each possible entity pair are concatenated and passed to the classification layer, which predicts the pair’s relation type. Cross-entropy loss is used for updating the model weights. PURE performs sentence-level extraction, assumes a single mention for each entity in each instance, and is designed for unidirectional relations. In contrast, the BioRED dataset contains full abstracts, multiple mentions of the same entity are common, and most relation types are bidirectional (e.g. Y is associated with X is equivalent to X is associated with Y). Therefore, we made several key updates to the model:

- **Directionality**: we removed subject and object designations to render relations bidirectional. For a given entity pair ENTITY1, ENTITY2, we generated two embeddings: [ENTITY1, ENTITY2, ENTITY1 x ENTITY2], and [ENTITY2, ENTITY1, ENTITY2 x ENTITY1], each corresponding to the concatenation of two entity representations and their element-wise product. These concatenated embeddings are individually passed to the relation classifier. The loss is the sum of the cross-entropy losses of both relation representations. To address the bidirectionality during prediction, the logits of both representations are summed up.
- **Multiple mentions**: we tag multiple mentions of the same entity. Each entity mention has its own corresponding marker token. However, for prediction, we select the pair of mentions (one for ENTITY1 mentions and one for ENTITY2 mentions) that best helps with classification. Our intuition is that not all mentions are important in identifying the relation, and may introduce unnecessary noise for the model. We take the dot product of each mention pair, which represents the importance of each mention pair to classifying the relation. We take the mention pair with the highest dot product (i.e. max pooling) and use it as the final relation representation for a given entity pair.
- **Entity type markers**: we also remove the distinction between different entity types for our marker tokens; i.e. instead of using [ENTITY- GENE] as a marker token, we only used [ENTITY]. Our initial experiments showed that including the entity type information in the marker token was not helpful for relation classification.

We utilized BioMedBERT as the base pretrained model and fine-tuned it with specified hyperparameters tuned using grid search: epochs (5), learning rate (3e-05), batch size (32), and optimizer (Adam). We selected BioMedBERT over BioLinkBERT as the pretrained model as BioMedBERT produced better performance during initial experiments. Additionally, to improve model robustness, we use projected gradient descent attacks [60] during training. After the model’s weights are updated using the combined loss, we perturb the token embeddings three times, adding noise, and train the model to correctly classify relations using the perturbed input.

### Novelty detection

We used a similar approach for ND task with two notable changes: (i) we did not include negative examples for training (as the input entity pairs already have an identified relation) and (ii) we used a different entity representation. Instead of picking the best pair of entity mentions, we weigh all mentions based on their importance for the ND task by using logsumexp pooling [61], a smooth version of max pooling, for all mentions of the entities in the entity pair. This generates a single vector for each entity, which we concatenate to obtain the final relation representation. Our intuition is that some mentions are more important than others; only in this case, we still consider all mentions as possibly contributing to the novelty prediction task. Similar to the RE model, we performed hyperparameter tuning using grid search. We trained the final models with the following hyperparameters: epochs (4), learning rate (2e-5), batch size (32), and optimizer (Adam).

## Results

### Development set results

#### Named entity recognition

The NER performances with BERT-based token classification and PURE span classification models are shown in Table 4. Scores include the NER post-processing step, described above. The token classification model demonstrated higher performance across most entity types as indicated by the F_1_ score. The span prediction model outperforms the token classification model on cell line entities. Overall, Species obtains the highest score on all metrics when only the BioRED dataset is used for training, while the performance is lowest for Disease and Cell Line entities.

**Table 4.** NER performance on the development set for the models trained on the BioRED dataset

	BERT-based token classification			PURE span classification		
Entity type	Precision	Recall	F_1_	Precision	Recall	F_1_
Gene	95.00	91.86	93.41	93.79	92.20	92.99
Disease	86.70	86.04	86.37	84.03	84.95	84.49
Chemical	89.24	93.50	91.32	89.03	92.57	90.77
Species	97.46	97.71	97.59	96.73	97.96	97.35
Variant	88.98	87.14	88.05	86.53	87.97	87.24
Cell line	82.35	84.00	83.17	90.91	80.00	85.11
**All**	**91.25**	**90.92**	**91.09**	**89.99**	**90.58**	**90.29**

Because of its higher overall performance, we opted to train the BERT-based token classification model on the combined dataset. Table 5 shows the results when all nine NER datasets are used for training. When all nine NER datasets are used, overall performance metrics increase by more than 2 percentage points, demonstrating the effectiveness of additional training data. For Species, the performance is slightly lower due to precision loss (−0.09 points). We obtained the most significant increases for Cell Line and Variant, two least frequent entity types in the BioRED dataset, particularly in precision (about 15 and 9 percentage points, respectively). With this increase, Variant performance surpasses that of the Species type.

**Table 5.** NER performance on the development set for the token classification model trained on all nine NER datasets

Entity type	Precision	Recall	F_1_
Gene	96.09	93.64	94.85
Disease	90.15	88.88	89.51
Chemical	93.20	92.75	92.95
Species	95.82	99.24	97.50
Variant	97.92	97.51	97.71
Cell line	97.78	88.00	92.63
**All**	**94.05**	**93.01**	**93.53**

To determine whether the performance difference between the token classification model that uses only BioRED and that uses all nine datasets was statistically significant, we performed bootstrap resampling, wherein we sampled 100 abstracts with replacement 1000 times. We calculated the overall F_1_ scores of these samples and compared the differences in scores. We found statistically significant difference between the performance of these models (mean difference: 1.54; 95% CI: 0.61, 2.85).

#### Entity linking

Table 6 shows the EL performance with PubTator 3.0 and ResCNN using predicted named entities as input as well as gold entities. PubTator 3.0 works well for Species entities but is less successful with other entity types. Using ResCNN-based EL models for Disease and Chemical entities improves EL performance for these entities. The improvement is especially significant for Disease entities (more than 9 percentage points) and less so for Chemical entities (about 1.5 points). Using gold entities generally leads to minor improvements, except in the case of Chemical type, where the difference is larger (more than 9 percentage points), which indicates that improving chemical entity recognition further could have significant impact in downstream tasks.

**Table 6.** EL performance based on predicted entities, along with the performance when gold entities are provided. All scores are based on the development set

	PubTator 3.0-only			+ ResCNN			+ ResCNN(GOLD)		
	P	R	F_1_	P	R	F_1_	P	R	F_1_
Gene	86.87	78.90	82.69	86.87	78.90	82.69	87.59	79.36	83.27
Disease	78.31	80.81	79.54	87.11	90.41	88.73	86.70	90.99	88.79
Chemical	87.37	78.64	82.78	83.19	85.45	84.30	91.93	93.18	92.55
Species	99.12	99.12	99.12	99.12	99.12	99.12	100.00	99.12	99.56
Variant	66.10	56.12	60.70	66.10	56.12	60.70	66.67	57.55	61.78
Cell line	85.00	77.27	80.95	85.00	77.27	80.95	82.61	86.36	84.44
**Total**	**83.50**	**78.65**	**81.00**	**85.37**	**82.42**	**83.87**	**87.20**	**84.46**	**85.81**

Table 7 shows the impact of augmenting the BioRED training set with external EL datasets (NCBI Disease [23] and BC5CDR [25]) and initializing token embeddings from different pretrained models (BioMedBERT [22] and BioLinkBERT [31]) for ResCNN training. The results show that additional training data consistently enhances the EL performance. Meanwhile, in terms of initial vector representations, BioMedBERT embeddings perform better with the BioRED training set, while BioLinkBERT outperforms BioMedBERT with additional dataset, although the differences are relatively minor. However, BioLinkBERT embeddings outperform BiomedBERT when it comes to using additional training set. To determine whether there is statistically significant difference in performance of models trained on BioRED and those trained with the additional datasets, we used McNemar’s test, which showed that the performance differences were statistically significant at 99% significance level.

**Table 7.** Evaluation of the impact of using additional datasets and different token embedding initializations on ResCNN-based EL models on the 100 samples of the BioCreative development set

Entity type	Embedding	Training set	Acc@1	Acc@5	Acc@10	Acc@20
Disease	BioMedBERT	Original	79.95	88.65	90.24	93.14
		+NCBI&CDR^a^	83.11	89.77	92.35	93.67
	BioLinkBERT	Original	78.89	88.39	91.29	93.14
		+NCBI&CDR^a^	83.91	90.77	92.35	94.20
Chemical	BioMedBERT	Original	88.50	91.00	93.00	93.50
		+CDR^a^	92.50	95.50	96.50	96.50
	BioLinkBERT	Original	88.00	92.00	92.00	92.50
		+CDR^a^	93.50	95.00	96.00	97.50

a Indicates statistically significant difference from the model trained with original data (99% significance level).

#### Relation extraction

Table 8 shows the results of the PURE-based RE model when gold standard entities and entity IDs are used as the model input. The performance is highest for Positive Correlation and Cotreatment, although the latter only has a few instances in the development set. Among the most common relation types, Association lags Positive Correlation and Negative Correlation. There were no predictions for the other rare labels, Conversion and Drug Interaction. When the relation types are ignored (i.e. binary relation classification), the model performance is 82.24 precision, 74.45 recall, and 78.15 F_1_ score, suggesting that distinguishing relation types is challenging for the model.

**Table 8.** RE model performance on the development set using gold standard entities and entity IDs

Entity type	Precision	Recall	F_1_
Association	67.43	54.32	60.17
Positive correlation	69.80	74.92	72.27
Negative correlation	68.54	69.71	69.12
Comparison	100.00	33.33	50.00
Bind	62.50	55.56	58.82
Conversion	0.00	0.00	0.00
Cotreatment	100.00	57.14	72.73
Drug interaction	0.00	0.00	0.00
**All**	**68.60**	**62.10**	**65.19**

Table 9 shows the results of the RE model when the predicted entities are used as model input (i.e. end-to-end RE pipeline). There is a 19 percentage point drop in F_1_ score (about 17 point drop in precision and 20 point drop in recall), indicating that errors in the previous two tasks significantly impact RE performance. The performance drop is similar for three relation types that occur in substantial numbers (Association, Positive Correlation, and Negative Correlation). About half of the predicted relations are incorrect; within these erroneous relations, 23.6% are incorrectly predicted as another relation type while 76.4% are due to non-related entities.

**Table 9.** RE model performance on the development set using predicted entities and IDs

Entity type	Precision	Recall	F_1_
Association	48.55	36.85	41.90
Positive correlation	55.83	48.62	51.97
Negative correlation	52.10	50.88	51.48
Comparison	100.00	33.33	50.00
Bind	100.00	33.33	50.00
Conversion	0.00	0.00	0.00
Cotreatment	37.50	21.43	27.27
Drug interaction	0.00	0.00	0.00
**All**	**51.48**	**41.87**	**46.18**

#### Novelty detection

Table 10 shows the performance of our PURE-based ND model. When the gold standard relations are known, the model predicts the novelty of about 80% of the relations accurately. Using gold standard entities and IDs and assessing the accuracy of the predicted relations and their novelty, the performance is about 10 percentage points lower than predicting relations alone (55.71 F_1_ score vs 65.19 F_1_ in Table 8). Lastly, in the end-to-end pipeline (NER-EL-RE-ND) where the model input consists only of the abstract text, there is about a 7 percentage point drop, compared to predicting relations only (38.89 F_1_ vs 46.18 F_1_ in Table 9).

**Table 10.** ND model performance on the development set

Input	Precision	Recall	F_1_
Gold standard relations	81.86	81.86	81.86
Gold standard entities	58.13	53.48	55.71
Abstract text only	43.34	35.25	38.89

### Test set results

We also generated predictions on the test set using RE and ND models trained on the combination of the training and development sets. This setting corresponds to Subtask 1 of the BioRED Track and uses gold standard entities and IDs. Table 11 shows the performance of these models on the test set. Compared to our shared task system [14], the enhanced RE model yields about 3 percentage points higher F1 score (55.61 vs 52.76) and the end-to-end RE + ND model increases F_1_ score by about 2 percentage points (41.66 vs 39.71). Our RE model performed best on relations between Chemical and Gene entities, obtaining an F_1_ score of 64.07. The lowest performance was on Chemical/Variant relations (37.03 F_1_). These results are likely due to abundance of Chemical/Gene relations and scarcity of Chemical/Variant relations in the training and development set.

**Table 11.** RE and ND performance on the test set using gold standard entities and entity IDs

	Precision	Recall	F_1_
RE	56.72	54.54	55.61
RE + ND	42.48	40.86	41.66

## Discussion

We enhanced our shared task system by including additional datasets in training and extensive hyperparameter tuning. As the system involves a pipeline approach, errors in earlier stages of the pipeline can propagate, leading to lower performance in later steps. By improving the performance in NER and EL, we were able to observe an improvement in downstream tasks of RE and ND. Table 12 shows a side-by-side comparison of the evaluation results of our best shared task submission [14] and our current system on the development set, which shows substantial improvement in each step of the pipeline. We note that the test set results can only be evaluated via CodaLab, which only focuses on the RE and RE + ND tasks; therefore, we are unable to assess the performance difference in NER and EL on the test set. As noted above, the improvement in RE and RE + ND performance on the test set was about 3 and 2 percentage points, respectively.

**Table 12.** Comparison with our previous shared task results

	Previous F_1_	New F_1_	Change
NER	90.44	93.53	+3.09
NER + EL	74.14	83.87	+9.73
NER + EL + RE	30.51	46.18	+15.67
NER + EL + RE + ND	23.96	38.86	+14.90

While the main architecture of our best NER model did not change, hyperparameter tuning and the inclusion of multiple NER datasets improved the results, which underscores the importance of large, annotated datasets and hyperparameter tuning for deep neural network models. We improved our EL performance by adopting a more accurate entity linker (i.e. PubTator 3.0 [16]) compared to previous linkers we used (BERN2 [54] and PubTator Central [46]) and training specialized CNN models for Disease and Chemical entities. As the performance of PubTator 3.0 was relatively low for these entity types and very often they served as relation arguments, they were considered as the entity types that could benefit most from specialized models. Incorporating additional EL datasets to the model training was also found to be beneficial. Using PubTator 3.0 alone led to an F_1_ increase of about 7 percentage F_1_ points (74.14 to 81), while CNN models led to another increase of about 3 points (81 to 83.87). Significantly, EL recall increased from 67.01 to 82.42, indicating that substantially more entities were linked to their corresponding identifiers, setting the stage for better RE and ND. One somewhat anomalous result relates to Variant entities, for which we obtain the highest NER performance (97.71 F_1_) but the lowest EL performance (60.70), which is especially surprising, because PubTator 3.0, which we used for variant EL, reports very high performance for this entity type (98.48 F_1_) [16]. A preliminary analysis suggests that there may be some differences in how the same variants are normalized in the ground truth data versus by PubTator 3.0, and in the EL evaluation in the shared task versus the PubTator 3.0 study.

As we expected, performance improvement in NER and EL increased the performance of RE and ND models, even though these models did not change from our shared task system, except for additional hyperparameter tuning. We note that, concurrent to our work, Lai *et al*. [35] incorporated additional RE datasets to improve RE performance on the BioRED dataset; so, the approach we used for NER and EL can further be extended to RE. There are no similar datasets for the ND task, so other approaches could be explored for ND, such as leveraging the abstract structure for novel information (i.e. novel information is less likely to be in introductory sections of the abstract).

We are unable to compare our enhanced pipeline with the results of the other shared task systems. Here, we compare our NER + EL + RE results with Pubtator 3.0 [16], which was officially released after the shared task (Table 13). Note that Pubtator 3.0 does not perform ND. The PubTator 3.0 yields 52.87 precision, 40.36 recall, and 45.77 F_1_ score on the development set. This is on par with our system; PubTator performs a bit better in precision (1.39 points), while our model performs slightly better in recall (1.51 points) and F_1_ score (0.41 points). More specifically, PubTator 3.0 performs better on Association and Negative Correlation relations, while our model shows better performance on Positive Correlation and Binding. The performance difference between PubTator 3.0 and our end-to-end RE model is statistically significant (McNemar’s test, 99% significance level).

**Table 13.** End-to-end RE performance using Pubtator 3.0

Entity type	Precision	Recall	F_1_
Association	56.13	38.63	45.76
Positive correlation	45.23	40.30	42.63
Negative correlation	58.11	49.71	53.58
Comparison	36.36	66.67	47.06
Bind	50.00	11.11	18.18
Conversion	0.00	0.00	0.00
Cotreatment	66.67	28.57	40.00
Drug interaction	0.00	0.00	0.00
**All**	**52.87**	**40.36**	**45.77**

Overall, RE remains challenging on the BioRED dataset. Low performance of end-to-end RE systems can be attributed to several factors. First, even though there are eight relation types, the great majority of the relations belongs to three types (Association, Positive Correlation, and Negative Correlation), and there are few examples of other relation types. The most common of these (Association) is particularly heterogeneous, and our models had modest performance on this type compared to correlation types. Our model (and PubTator 3.0) did not yield positive predictions for two rare types. Given that most biologically relevant relations are mechanistic (causal), the lack of directionality in BioRED relations could be problematic for downstream uses. The BioRED relations are document-level, which is a more challenging setting than sentence-bound RE, although arguably more relevant. More comprehensive RE datasets reflecting the complexity of the biological processes and better-performing RE models are needed to enable practical use of biomedical RE models and tools.

### Error analysis

To shed more light on the shortcomings of our pipeline, we performed error analysis of our NER, EL, and ND predictions on the development set. NER predictions from the BERT-based model were categorized as exact matches (boundaries and entity type match, *n* = 3336), partial matches (boundaries partially match, *n* = 67), and complete misses (*n* = 132). Among the complete misses, 92% belonged to Disease (*n* = 58), Gene (*n* = 30), and Chemical types (*n* = 34). Overall, Disease entity type had most mismatches. Future work could focus on improving the performance of Disease and Gene entity types in particular.

We analyzed the errors made by the ResCNN-Disease model. We adopted the error types from BioSyn [44] in our analysis: Incomplete Synset (input mention differs significantly from the synonyms of the identifier), Contextual Entity (mention and synonym are identical but have different identifiers), Overlapped Entity (word overlap between the mention and the predicted candidate), Abbreviation (abbreviation cannot be resolved), Hypernym/Hyponym (mention and the concept identifier have hypernym/hyponym relation). Table 14 shows the examples and frequency of the error cases. Almost half of the errors are due to hypernym-hyponym relations, followed by overlapped entity sets. Hypernym-hyponym errors could be considered less severe (especially if the hyponym mention is mapped to a hypernym concept). Incomplete synonym sets could be addressed through better modeling of the semantic similarity between the mentions and the synonyms. Contextual entity errors are challenging because ResCNN only takes the named entity mention as input, not the surrounding context, so it may favor an identifier that has more surface similarity over an identifier that is a better conceptual match.

**Table 14.** EL error examples and frequency counts on the development set

Error type	Input mention	Predicted name	Gold concept name	Frequency
Incomplete Synset	*hyper locomotion*	*neurologic locomotion disorders*	*hyperkinesis*	2 (3.4%)
Contextual Entity	*colon cancer*	*colon cancer*	*colorectal neoplasms*	5 (8.6%)
Overlapped Entity	*postoperative analgesia*	*congenital analgesia*	*pain postoperative*	9 (15.5%)
Abbreviation	*veds*	*ved*	*ehlers danlos syndrome*	5 (8.6%)
Hypernym	*dyskinesia*	*dyskinesia*	*dyskinesia drug induced*	12 (20.7%)
Hyponym	*familial melanoma*	*melanoma*	*familial melanoma*	20 (34.5%)
Others	*hyperthermia*	*hyperthermia*	*fever*	5 (8.6%)

In RE, 43.45% of precision errors involved type errors; in other words, the model was able to correctly identify that the two entity pairs are related but assigned the incorrect relation type. The rest of the errors are false-positives involving non-related entities. On the other hand, 31.98% of the recall errors are due to incorrect relation type assignment, while the majority (68.02%) is due to the model failing to identify relationships between entities. Relations involving variant types obtained the lowest recall; 39.09% of relations where at least one of the entities is a variant were missed by our model, including all variant–variant relations (10 instances). Our model missed 27.79% and 24.74% of relations involving diseases and genes, respectively. Chemical relations obtained the highest recall, and only 17.9% of relations involving at least one chemical was missed by the model.

The majority of relation type confusion cases occurred between Association, Positive Correlation, and Negative Correlation. There were only four instances of type confusion involving other relation types. Specifically considering the most common relation type (Association), we found that 7.71% and 3.62% were identified as Positive Correlation and Negative Correlation, respectively, while 35.27% were missed by the model completely. Interestingly, it was significantly more common for Positive Correlation relations to be incorrectly labeled as Association relations compared to Negative Correlation relations. These results indicate the need to focus on associative relations in future work.

## Conclusion

We presented an enhanced end-to-end pipeline for biomedical RE and ND. Compared to our BioCreative VIII BioRED Track submission, our pipeline demonstrates substantial performance improvement across all four tasks of the pipeline (NER, EL, RE, and ND). In particular, enhancements on our NER and EL methods including the use of additional datasets improved the performance of the target tasks of RE and ND, as well. Despite considerable increases in our pipeline performance and being on par with PubTator 3.0, there is much room for improvement, especially in RE and ND tasks.

The BioRED dataset and the BioCreative VIII BioRED Track are significant steps in expanding biomedical RE from a few relation types to a more comprehensive set of relevant relation types and practical use cases. However, our work also highlights some important challenges; e.g. relations are skewed toward a few types and may not be sufficiently specific, and document-level relation formulation, while flexible, also presents difficulties in interpretation of the relations and predictions. Further enhancements to the dataset could facilitate more accurate and useful systems for information extraction from the biomedical literature.

## Data Availability

The BioRED track dataset and other challenge materials are available at https://ftp.ncbi.nlm.nih.gov/pub/lu/BC8-BioRED-track/. The code and models generated in this study are available at https://github.com/janinaj/e2eBioMedRE/.
